# Disseminated Aspergillus flavus following septic arthritis in an immunocompetent patient: a case report

**DOI:** 10.1186/1756-0500-7-709

**Published:** 2014-10-09

**Authors:** Vivek Tiwari, Kavin Khatri, Shah Alam Khan, Devajit Nath

**Affiliations:** Department of Orthopaedics, All India Institute of Medical Sciences, New Delhi, 110029 India; Department of Pathology, All India Institute of Medical Sciences, New Delhi, India

**Keywords:** Aspergillosis, *Aspergillus flavus*, Disseminated, Immunocompetence, Septic arthritis, Knee, Tuberculosis

## Abstract

**Background:**

Aspergillosis is a rare cause of osteomyelitis and septic arthritis. Aspergillus osteomyelitis is a debilitating infection affecting both immunocompromised and immunocompetent patients. It is associated with a high incidence of morbidity and mortality. Infection with *Aspergillus flavus* species in the knee has been very rarely seen in the past.

**Case presentation:**

We present a case of septic arthritis of the knee in an Indian patient secondary to infection with *Aspergillus flavus,* which was earlier managed as a case of tuberculosis based on the endemicity of the condition, later leading to disseminated aspergillosis. There was no clinical feature or investigation suggesting immunocompromised state. Following knee arthrotomy, *Aspergillus flavus* was isolated and patient was subsequently managed with antifungals leading to recovery after three months.

**Conclusions:**

Disseminated aspergillosis can mimic tuberculosis both clinically and radiologically. Though fungal infections affect joints rarely but they must always be ruled out to avoid later complications.

## Background

Aspergillus is widely distributed throughout the environment. The primary infection of a joint by an aspergillus species, however, is uncommon. Although, earlier it was reported to affect predominantly the immunocompromised patients [1], recent studies conclude that non-immunocompromised patients are also an important population at risk [2]. The infection usually spreads to the knee joint through haematogenous route from lungs. The immunocompetent population may have a history of preceding surgery or open fractures, or may have no prior surgical procedure or trauma [2]. Septic arthritis is a rheumatologic emergency as delay in treatment can lead to irreversible damage to the joint. Aspergillosis though a rare cause of septic arthritis is associated with a high incidence of morbidity and mortality [3]. We present a case of septic arthritis of the knee secondary to infection with *Aspergillus flavus* which later lead to disseminated aspergillosis.

## Case presentation

**Figure 1:**
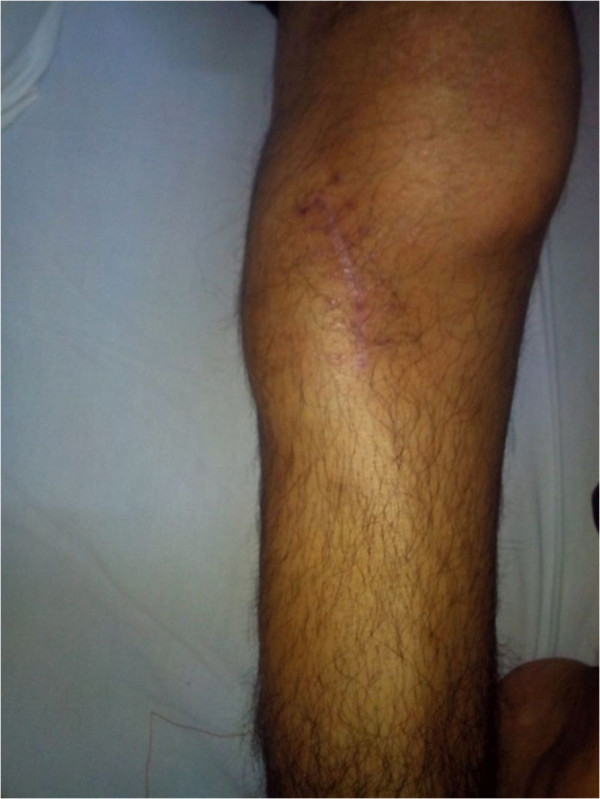
**Clinical photograph of the knee.** A smooth, soft, fluctuant, tender, well-defined swelling of size 7 × 5 cm on the anterolateral aspect of the right knee and proximal leg.

**Figure 2:**
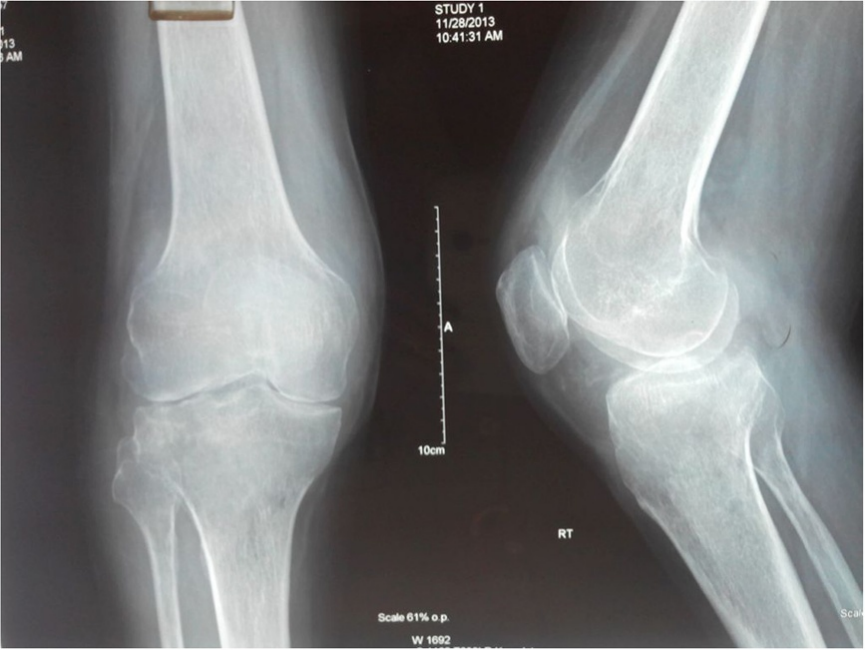
**Plain radiograph of right knee.** Plain radiograph of the right knee demonstrated mild peri-articular osteopenia with global reduction in joint space along with soft tissue density.

**Figure 3:**
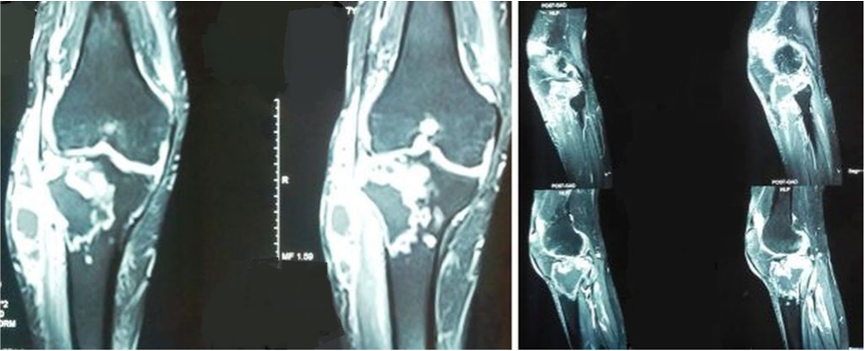
**Magnetic resonance imaging of right knee.** Magnetic Resonance Imaging of the right knee joint showing intra-osseous and extra-osseous abscesses with synovial inflammation involving knee joint and supra-patellar bursa.

**Figure 4:**
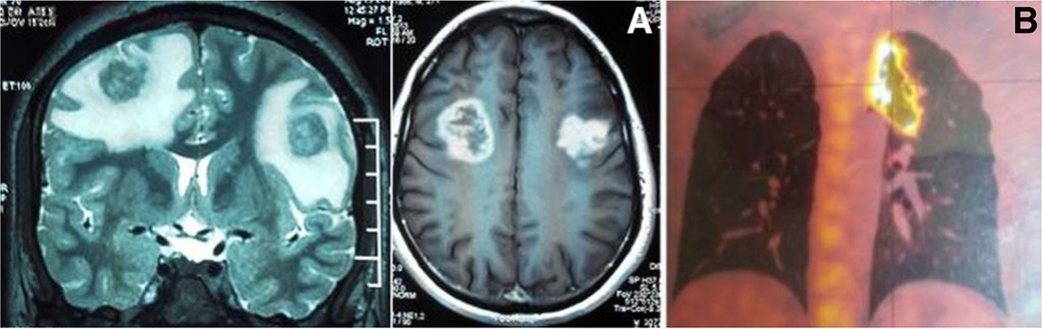
**Non contrast computed tomograph of brain and whole body positron emission tomography scan.** **A**- Non Contrast Computed Tomograph of the brain showing large lobulated lesions in bilateral frontal lobes likely representing fungal abscesses. **B**- Whole body Positron Emission Tomography Scan showing hypermetabolic cavitatory lesion in the upper lobe of left lung.

A 28-year-old Indian male patient presented with right knee swelling for one and a half year associated with severe pain and inability to bear weight on right lower limb. The onset of the swelling was associated with low grade continuous fever for six months which used to subside with anti-pyretics to recur again. Radiographs of the right knee did not reveal any significant joint or bony pathology. The patient was empirically advised to take oral amoxicillin-clavulanic acid 625 mg thrice a day along with low dose steroid 5 mg prednisolone once a day by a local practitioner for one month but there was no relief in knee pain or swelling. He was further subjected to chest radiography in which a cavitatory lesion was noticed in upper lobe of left lung. Tuberculosis is an endemic condition in India and suspecting the same he was prescribed anti-tubercular therapy (4 drug regimen - isoniazid, rifampicin, pyrazinamide and ethambutol). Within a week of starting antitubercular therapy, his fever subsided but at the same time he developed rashes all over his body associated with itching. The symptoms were attributed to pyrazinamide hypersensitivity reaction. The anti-tubercular drugs were stopped for five days and symptoms were managed with oral levo-cetrizine. Pyrazinamide was then excluded from regimen and he was asked to continue rest of the medication. He took antitubercular drugs for seven months but there was no decrease in the knee pain and swelling. Approximately seven months after the start of antitubercular treatment, he had an episode of generalised tonic clonic seizure which was managed with tablet levetiracetam. He was then advised to undergo magnetic resonance imaging (MRI) of the brain. It revealed frontal lobe masses bilaterally which were hyperintense on T2 and hypointense on T1, the right one measured 2.9 × 2.4 × 2.7 cm and the left one measured 2.2 × 2.2 × 1.9 cm. It was again presumed to be a manifestation of disseminated tuberculosis because of endemicity of tuberculosis in the region and its focus was considered to be from the knee joint and left lobe of the lung. Subsequently, amikacin and moxifloxacin were added in the anti-tubercular regimen suspecting it to be a case of multi-drug resistant tuberculosis. There was no change in the knee swelling, moreover a swelling appeared in the right proximal leg close to knee joint after ten months of antitubercular therapy. He again had two episodes of generalized tonic clonic seizure after eleven months of anti-tubercular therapy. Subsequently, phenytoin was added to his treatment for seizures along with antitubercular drugs. Keeping in mind drug interactions between rifampicin and phenytoin, serum phenytoin levels were done and thedrug dosage was adjusted. Following this he presented to the orthopaedic outpatient department of our institute with complaints of right knee pain and swelling extending to right leg. There was no prior history of any invasive procedure (biopsy or aspiration) done on the right knee.On examination, he appeared lethargic and was afebrile. His systemic examination including the chest was unremarkable. He had a smooth, soft, fluctuant, tender, moderately warm, well-defined swelling of size 7 × 5 cm on the anterolateral aspect of the right knee and proximal leg (Figure 1). The overlying skin was shiny. The local temperature was raised as compared to the contralateral knee joint. There was no neurovascular deficit.His total blood counts were in normal range with relative neutrophilia. Erythrocyte sedimentation rate by Westergren’s method was 90 mm/hour (normal range ≤ 20 mm/hour for males) and serum C-reactive protein level was 85 mg/litre (normal range < 6 mg/litre). The radiographs of the right knee demonstrated mild peri-articular osteopenia with global reduction in joint space along with soft tissue density around the knee joint (Figure 2).Contrast enhanced MRI of the right knee joint revealed infiltrative peripheral enhancing lesion, which was hyperintense on T2 and hypointense on T1 sequences, in the lateral tibial condyle with conglomerate, lobulated similar signal intensity lesion in the overlying soft tissues, likely representing intra-osseous and extra-osseous abscesses with synovial inflammation involving the knee joint and the supra-patellar bursa (Figure 3).Contrast enhanced MRI of the brain revealed large lobulated lesions, hyperintense on T2 and hypointense on T1, in bilateral frontal lobes with irregular heterogenous peripheral enhancement showing peripheral restricted diffusion and peri-lesional edema. Magnetic resonance spectroscopy showed elevated lactate levels in the lesions, likely representing fungal abscesses. There was slight increase in size of the lesion as compared to the previous scan (Figure 4A).Whole body positron emission tomography-computed tomography (PET-CT) scan demonstrated hypermetabolic cavitatory lesion in the upper lobe of left lung with associated ring enhancing lesions in the cerebrum (Figure 4B).

**Figure 5:**
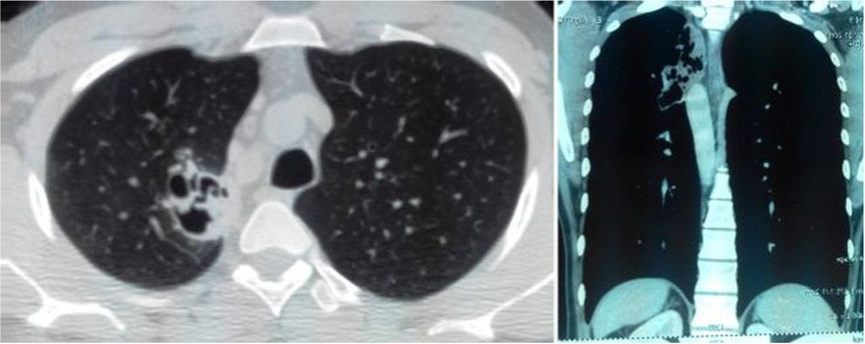
**Contrast enhanced computed tomograph of chest.** Contrast Enhanced Computed Tomograph of chest showing an irregular moderately thick walled cavitatory lesion in the left upper lobe postero-medially with nodularity along the inner wall of the cavity.

Contrast enhanced computed tomography (CECT) scan of the chest revealed an irregular, moderately thick-walled cavitatory lesion in the upper lobe of left lung with nodularity along the inner wall of the cavity at places and a rounded well-defined intra-cavitatory nodule along upper part of the cavity suggesting presence of disseminated aspergillosis (Figure 5). This could also be explained by formation of tubercular cavitation initially followed by aspergillosis development inside. Costal aspergillosis due to contiguous involvement of ribs from the pulmonary focus has also been described in literature [4]. It was ruled out as the computed tomography (CT) scan was done using both lung and bone windows, and also due to absence of pleuritic pain, localised tenderness and swelling.

**Figure 6:**
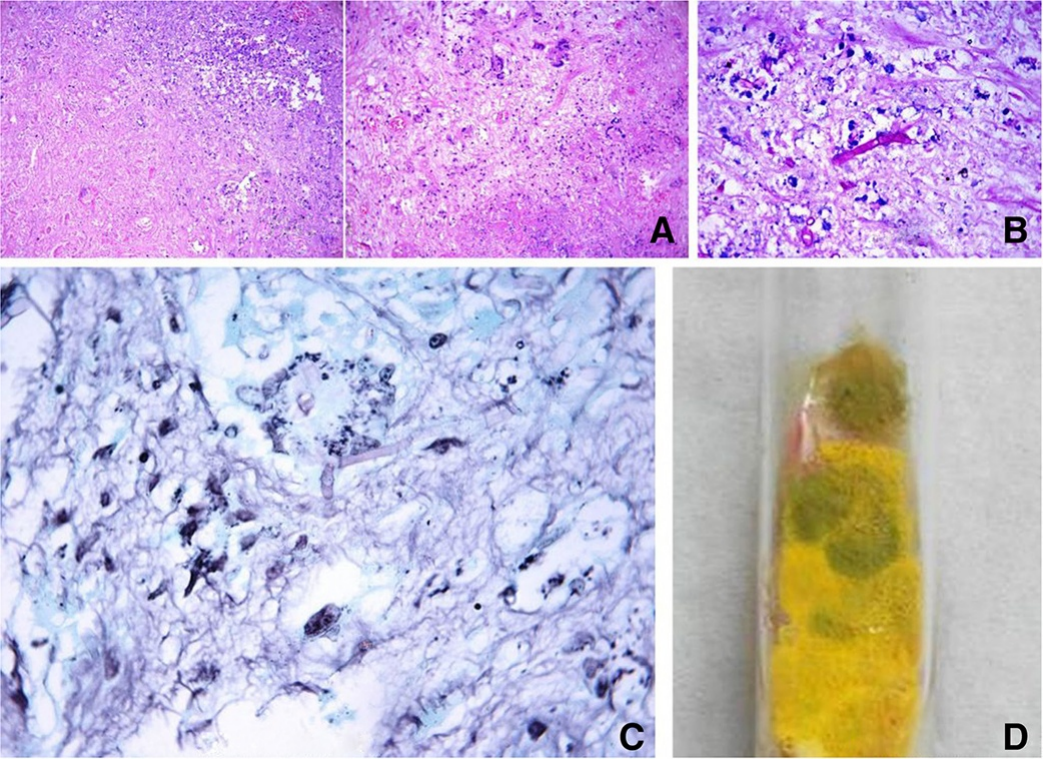
**Histopathology and growth on culture medium.** **A**- Haematoxylin & Eosin stained slides from the proximal tibia showing predominantly necrotic tissue with acute inflammatory exudate and focal giant cell formation. **B**- Periodic acid Schiff stained tissue section revealed stout septate fungal hyphae of Aspergillus species. **C**- Methenamine silver stained tissue section also showed septate hyphae of Aspergillus species. **D**- The specimen cultured growth of *Aspergillus flavus* on fungal culture medium.

Biopsy was done from the right proximal tibia and the histopathology report revealed necrotic tissue with acute inflammatory exudate and focal giant-cell formation suggestive of osteomyelitis (Figure 6A). Presence of knee joint swelling along with the above findings suggested contiguous involvement of knee from the osteomyelitic focus causing septic arthritis. The cytopathology report from the intra-articular fluid demonstrated mainly acute inflammatory exudate and necrosis along with occasional giant-cells. The stain for acid fast bacillus was negative. Periodic acid schiff (PAS) stained tissue section showed stout septate fungal hyphae of Aspergillus species (Figure 6B). Methenamine-silver-stained tissue section also showed septate hyphae of Aspergillus species (Figure 6C). Galactomannan test was also positive in the intra-articular fluid suggesting presence of Aspergillus. The growth of *Aspergillus flavus* was seen on the fungal culture medium (Figure 6D).

A diagnosis of disseminated aspergillosis was made following the above investigations. Subsequently, he was put on oral voriconazole and intravenous amphotericin B (for 15 days), as this combination therapy is reported to be effective in invasive aspergillosis [5], along with intravenous dexamethasone and knee arthrotomy was done. Femoral and tibial articular surfaces were found to have erosions with geographic hyper-pigmented lesions. Dead and necrotic tissue was debrided from the knee joint and thorough wash done. Biopsy samples sent from the bone as well as the synovium during arthrotomy confirmed the presence of *Aspergillus flavus.* Synovial fluid sent for culture also growed colonies of *Aspergillus flavus* on the fungal culture medium confirming it as a case of proven aspergillus arthritis. He had no recurrence of fever or seizures following the surgery and the knee swelling and pain subsided after 3 months of antifungal treatment with oral voriconazole.

Staphylococcus is the most common organism implicated in septic arthritis [6]. Fungal infections, especially Candida and Aspergillus, though rare have been found to occur more commonly in diabetics and immunosuppressed persons [7]. They lead to an opportunistic infection once there is decrease in the immunity of the body. As far as aspergillosis is concerned, it involves lungs, brain, kidneys, liver and gastrointestinal tract in majority of the cases [8]. Aspergillosis has also a predisposition for vertebrae and ribs [8, 9]. The hip joint is most commonly involved joint followed by knee, wrist and ankle among the cases of articular aspergillosis [2]. Aspergillus genus includes more than 35 species of saprophytic moulds among which *Aspergillus fumigatus* is the most common pathogenic organism [10].

The diagnosis of Aspergillus osteomyelitis often requires more stringent measures than required in cases of bacterial osteomyelitis. The general accepted norm in its diagnosis includes repeated culture of the fungus from the diseased site along with histo-pathological examination demonstrating inflammatory exudates, giant-cell reaction and numerous branching fungal hyphae. These findings along with the clinical manifestations of the disease help in arriving at the diagnosis [11]. In cases of septic arthritis, the organism can be isolated from the synovial fluid and the total leukocyte cell counts are generally above 5000/mm3 associated with a relative neutrophilia. Aspergillus grows very fast and the cultures are usually visible in 2 to 4 days, though in some cases it may require a longer incubation period. The fungus is found extensively in the environment and infects almost each one of us but the clinical manifestations appear more commonly in immunocompromised cases. Although recent studies report that Aspergillus osteomyelitis can be seen in immunocompetent patients also, septic arthritis in such patients is still believed to be a rare entity and its pathogenesis is not known [2, 12]. In our case, neither the clinical features nor the investigation reports suggested any sign of immunosuppression.

The treatment of Aspergillus arthritis includes surgical drainage along with administration of antifungal agents like amphotericin B and voriconazole. There is, however, no consensus on the use of amphotericin B or voriconazole in the treatment of articular aspergillosis [13]. There is risk of nephrotoxicity with the use of amphotericin B so its maximum dose and duration should be stringently regulated. Voriconazole can be used in both intravenous and oral dosage form with lesser side effects as compared to amphotericin B.

## Conclusions

Disseminated aspergillosis can mimic tuberculosis both clinically and radiologically. In the index case the empirical treatment for tuberculosis was started based on the endemicity of the condition. Blood and radiological investigations were not carried out to reach a diagnosis and hence the patient had developed disseminated aspergillosis. Though fungal infections affect joints rarely, they must always be ruled out to avoid later complications.

## Consent

Written informed consent was obtained from the patient for publication of this Case Report and any accompanying images. A copy of the written consent is available for review by the Editor-in-Chief of this journal.

## Abbreviations

MRI: magnetic resonance imaging
PET-CT: positron emission tomography-computed tomography
CECT: contrast enhanced computed tomography
CT: computed tomography
PAS: periodic acid schiff.
